# Non-Cardiogenic Pulmonary Edema Due to Administration of Atosiban

**DOI:** 10.7759/cureus.36799

**Published:** 2023-03-28

**Authors:** Alexandros Dalkalitsis, Athanasios Zikopoulos, Alexandros Katrachouras, Ioanna Samara, Fani Gkrozou

**Affiliations:** 1 Obstetrics and Gynaecology, University Hospital of Ioannina, Ioannina, GRC; 2 Obstetrics and Gynaecology, Royal Cornwall Hospital, Cornwall, GBR; 3 Cardiology, University Hospital of Ioannina, Ioannina, GRC

**Keywords:** intensive care unit, preterm labor, non-cardiogenic, pulmonary edema, atosiban

## Abstract

We report the case of a pregnant woman, treated with atosiban for premature labor, who developed non-cardiogenic pulmonary edema. She corresponded initially to oxygen supplementation and furosemide administration to induce diuresis but the onset of preterm contractions combined with aggravation of respiratory failure led the patient to a cesarean section, and subsequently to the intensive care unit where she remained intubated for 24 hours. In this case report, we emphasize the importance of distinguishing between two types of pulmonary edema: cardiogenic and non-cardiogenic. The instant separation between these two categories, most of the time with transthoracic echocardiography while the patient is on early support of ventilation, increases the optimum outcome for the patient.

## Introduction

Acute pulmonary edema is relatively uncommon in pregnancy. The incidence rate varies from 0.08% to 0.5% [1]. It can be broadly classified into cardiogenic and non-cardiogenic, according to the underlying cause.

Cardiogenic pulmonary edema is due to structural or functional cardiac dysfunction. The main mechanism is increased left atrium pressure (LAP), usually due to increased left ventricular end-diastolic pressure, leading to high pulmonary capillary pressure. Consequently, increased fluid transfer out of capillaries is provoked. Common causes in pregnancy are peripartum cardiomyopathy, valvulopathy, myocarditis, Takotsubo syndrome, acute coronary syndrome, hypertrophic and dilated cardiomyopathy, congenital heart disease, and arrhythmias [2].

Non-cardiogenic pulmonary edema is characterized by the accumulation of fluid in alveoli without hemodynamic evidence of high pulmonary capillary wedge pressure [3]. The underlying pathophysiologic mechanism is the disruption of endothelial and/or epithelial layers, which leads to increased alveolar-capillary barrier permeability [3].

## Case presentation

A 46-years old pregnant woman (G1P0) was admitted for threatened preterm labor at 29 weeks of gestation with dichorionic/diamniotic twins after in vitro fertilization (IVF). She had no relevant medical, obstetric, or family history. Regarding the present pregnancy, the patient was treated for gestational diabetes mellitus with nutritional therapy and for hypothyroidism with T4 100 μcg daily. Additionally, she was on acetylsalicylic acid (Salospir 150 mg) and vaginal progesterone to manage short cervical length (22 mm at 22 weeks of gestation).

At admission, cardiotocography showed regular uterine activity without signs of fetal distress. Vital signs were within normal limits (BP: 124/76 mmHg, pulse: 85 bpm, SaO2: 98%, temperature: 36.2 °C), and there were no signs of chorioamnionitis. Blood sample analysis was within normal limits (Table 1).

**Table 1:** Evaluation of labs on admission day

Parameter	Result	Normal range
White blood cell count (×10^3^/mm^3^)	10.87	3.8-11.8
Hematocrit (%)	36.5	33-47
Hemoglobin (g/dL)	11.7	11.2-14.5
Platelet (×10^9^/L)	289	150-400
C-reactive Protein (mg/L)	1	<8
Glucose	75	70-125
Alanine transaminase (unites/L)	27	10-35
Aspartate transaminase (unites/L)	16	10-35
Urea nitrogen (mg/dL)	13	11-54
Creatinine (mg/dL)	0.77	0.6-1.2

The fetal ultrasound examination was normal while the cervical length was 8 mm.

She was administered betamethasone two doses of 12 mg intramuscularly 24 hours apart, to reduce the incidence and severity of respiratory distress syndrome and mortality in the offspring, and magnesium sulfate (4g intravenous loading dose over 20 min followed by a 1g/hour infusion) to reduce the incidence of cerebral palsy and severe motor dysfunction. An induction dose of atosiban, as tocolytic treatment, was administered intravenously (6.75 mg), followed by 18 mg/h for three hours and 6 mg/h resulting in cessation of uterine contractions. In addition, she was also administered ceftriaxone 1g once daily (QD), metronidazole 500 mg three times a day (TDS), and clarithromycin 500 mg twice daily (BD), as per the local protocol of threatened preterm labor. The following day, the patient was feeling well and administration of atosiban was continued according to the manufacturer’s instructions. In addition, a vaginal silicone cerclage pessary (Arabin®, Dr Arabin, Witten, Germany) was inserted.

Two days after admission, the patient complained about shortness of breath. Pulse oximetry showed oxygen desaturation (SaO2: 92%), which was confirmed by arterial blood gas analysis (Table 2).

**Table 2:** Arterial blood gas exams on Day 2

Parameter	Result	Normal range
Ph	7.43	7.35-7.45
PO_2 _(mmHg)	80	90-100
PCO_2_ (mmHg)	37	35-45
Bicarbonate (mEq/L)	25	22-26
SaO_2_ (%)	92	>95

**Figure 1:**
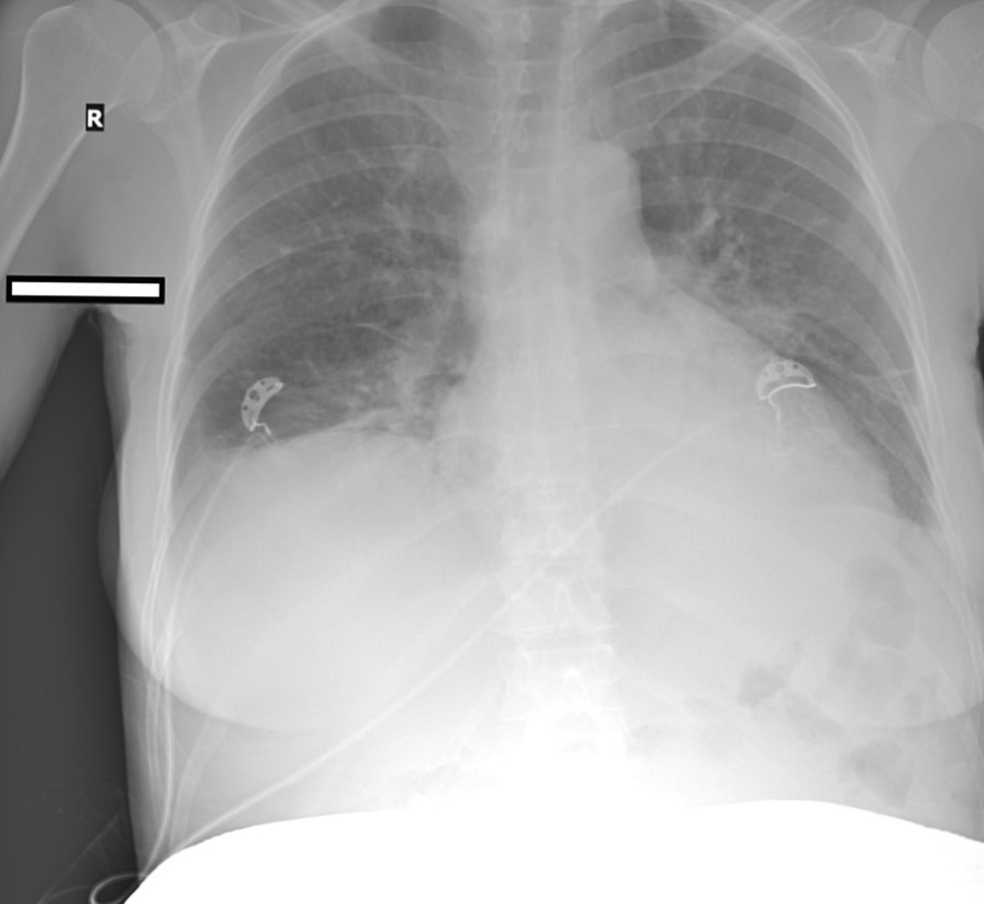
X-ray of the chest

Blood pressure was 110/82 mm/Hg and pulse rate 84 bpm. Initially, nasal oxygen was applied at 4 liters/min. Due to acute respiratory failure, urgent cardiology and pulmonology opinions were sought and low-molecular-weight (LMW) heparin was administered at therapeutic levels due to the high suspicion of pulmonary embolism (PE). Lung auscultation revealed left-sided crackles and decreased breath sounds in the left lung. Cardiac sounds were normal. The patient underwent compression ultrasonography (CUS) as an initial diagnostic test, which was negative for deep vein thrombosis (DVT). Additionally, chest X-ray (CXR) and computerized tomography pulmonary angiography (CTPA) was conducted as well (Figure 1).

Imaging showed a shadow in the right lower pulmonary lobe, attributed to a previous Covid-19 illness and signs of pulmonary edema. Transthoracic echocardiography revealed good left ventricular function with ejection fraction over 60%, good right ventricular function, no valvulopathies, normal filling pressures, and no pericardial effusion. The 12-lead electrocardiogram showed sinus rhythm, with no signs of ischemia or conduction abnormalities. Increase in oxygen supply was necessary at this time since percutaneous oxygen saturation did not rise above 91%. Atosiban was discontinued and two doses of 10 mg intravenous furosemide an hour apart led to clinical improvement.

The following day, the patient was stable and well up to the early evening, when she started developing contractions. Nifedipine 20 mg was administered in an effort to inhibit acute preterm labor. An hour later respiratory aggravation was observed with SaO2: 90%, 32 breaths per minute, and aggravated arterial blood gas values (Table 3).

**Table 3:** Arterial blood gas exams on Day 3

Parameter	Result	Normal range
Ph	7.47	7.35-7.45
PO_2 _(mmHg)	67	90-100
PCO_2_ (mmHg)	29	35-45
Bicarbonate (mEq/L)	20	22-26
SaO_2 _(%)	89	>95

Intravenous furosemide 20 mg was given twice and the patient was transferred to the intensive care unit. Noninvasive ventilation with high flow nasal cannula led to clinical improvement. During the night, the patient developed severe and regular contractions, which resulted in cervical effacement and an emergency cesarean section was performed under general anesthesia. Two fetuses of 1420gr and 1160gr were born and transferred to the neonatology ward for further assessment and care. An intrauterine balloon was inserted to avoid excess medical management with uterotonics since the patient suffered from pulmonary dysfunction.

The patient was kept intubated for 24 hours and remained in the intensive care unit with supplemental oxygen for an additional day. In turn, she was transferred to the maternity ward and oxygen therapy was discontinued.

## Discussion

Acute respiratory failure in pregnancy requiring mechanical ventilation affects approximately 0.1%-0.2% of pregnancies [4]. Our patient suffered acute pulmonary edema without presenting hypertension, as one of the most common causes of acute respiratory failure due to pulmonary edema is preeclampsia [1]. Other causes of non-cardiogenic pulmonary edema include tocolytic drug administration, sepsis, aspiration, iatrogenic causes (e.g. intravenous fluid overload), and amniotic fluid embolism [1]. In the above cases, the alveolar-capillary membrane becomes damaged, thereby increasing capillary permeability and altering forces controlling pulmonary interstitial fluid. In addition, fluid overload, as well as steroid medication, participate in the pathophysiology of non-cardiac pulmonary edema as well [1].

Clinical presentation of pulmonary edema due to tocolytic drugs has similar signs and symptoms with pulmonary edema of other causes. Dyspnea, tachypnea, hypoxemia, and crackles are typical features. The use of tocolytic beta-agonists (e.g. terbutaline) to inhibit preterm labor is associated with pulmonary edema [5,6]. This is more common in multiple gestation pregnancies and among pregnancies with maternal infection (e.g. urinary tract infection, pneumonitis) [7]. Nifedipine administration is associated with pulmonary edema as well; however, it is less frequent [1,8,9]. In addition, pulmonary edema has been reported to occur by administering magnesium sulfate. In this case, it is still uncertain whether this is due to the etiology itself or to volume overload. Pulmonary edema appears to be exacerbated by the administration of high doses of magnesium sulfate and/or a high intravenous infusion rate [10,11]. Atosiban which was also administered in our patient is a selective oxytocin-vasopressin receptor antagonist. The overall frequency of cardiovascular side effects is prominently fewer than any other tocolytic drug [12,13].

To the best of our knowledge, there are only three case reports in literature correlating atosiban with life-threatening non-cardiogenic pulmonary edema [14-16]. The mechanism by which pulmonary edema is caused is not fully clarified. However, we assume that the mechanism is related to other tocolytic drugs and possibly associated with increased capillary permeability in combination with fluid overload. Considering that betamethasone is probably not a major risk factor for pulmonary edema, administration of magnesium sulfate was approximately 72 hours prior to the event, and administration of nifedipine was more than 24 hours after the first episode of acute respiratory failure; it appears that a clear association between non-cardiogenic lung edema and atosiban was present in the precurrent case report.

## Conclusions

In conclusion, the application of atosiban should raise concerns about acute respiratory failure since pulmonary edema can be an uncommon side effect, especially when there is concomitant or previous administration of other tocolytic drugs (e.g. MgSO4, nifedipine) or fluid overload. This, however, should not inhibit its broad use, as its role in tocolysis is well-established and crucial, while its safety profile is better compared to other types of tocolytics.
